# Microglial Ultrastructure in the Hippocampus of a Lipopolysaccharide-Induced Sickness Mouse Model

**DOI:** 10.3389/fnins.2019.01340

**Published:** 2019-12-20

**Authors:** Julie C. Savage, Marie-Kim St-Pierre, Chin Wai Hui, Marie-Eve Tremblay

**Affiliations:** ^1^Axe Neurosciences, Centre de Recherche du CHU de Québec-Université Laval, Quebec, QC, Canada; ^2^Départment de Médecine Moléculaire, Faculté de Médecine, Université Laval, Quebec, QC, Canada; ^3^Division of Life Science, The Hong Kong University of Science and Technology, Kowloon, Hong Kong

**Keywords:** sickness behavior, lipopolysaccharide, neuroinflammation, microglia, phagocytosis, hippocampus, mouse, electron microscopy

## Abstract

Sickness behavior is a set of behavioral changes induced by infections and mediated by pro-inflammatory cytokines. It is characterized by fatigue, decreased appetite and weight loss, changes in sleep patterns, cognitive functions, and lost interest in social activity. It can expedite recovery by conserving energy to mount an immune response involving innate immunity. To provide insights into microglial implication in sickness behavior with special focus on cognitive and social impairment, we investigated changes in their ultrastructure and interactions with synapses using a toxemia mouse model. Adult mice were injected with 1 mg/kg lipopolysaccharide (LPS) or saline, and assayed for signs of sickness behavior. LPS treated mice displayed reduced activity in open-field tests 24 h post-injection, while social avoidance and weight gain/loss were not significantly different between treatment groups. Microglia were investigated using electron microscopy to describe changes in their structure and function at nanoscale resolution. Microglial cell bodies and processes were investigated in the hippocampus CA1, a region responsible for learning and memory that is often impacted after peripheral LPS administration. Microglia in LPS treated animals displayed larger cell bodies as well as less complex processes at the time point examined. Strikingly, microglial processes in LPS injected animals were also more likely to contact excitatory synapses and contained more phagocytic material compared with saline injected controls. We have identified at the ultrastructural level significant changes in microglia-synapse interactions shortly after LPS administration, which draws attention to studying the roles of microglia in synaptic rewiring after inflammatory stimuli.

## Introduction

Sickness behavior is a well-defined set of cognitive and behavioral adaptations recruited in response to bacterial or viral infection or peripheral increases in proinflammatory cytokines. It is characterized by fatigue, joint and muscle pain, coldness, and reduced appetite (Dantzer, 2009) accompanied by psychological, emotional, and behavioral disturbance (Dantzer et al., 2008). Sepsis is a life-threatening condition caused by the body’s immune response to infection. Massive increases in blood and serum levels of cytokines and chemokines can cause organ failure and breakdown of the blood-brain barrier (BBB). Septic-associated encephalopathy is a serious complication, with symptoms including cognitive impairment and seizures.

Peripheral macrophages are able to recognize viruses, bacteria, fungi, and other invading pathogens using cell-surface receptors which recognize pathogen and danger associated molecular patterns (PAMPs and DAMPs). Upon recognition of dangerous materials, these cell surface receptors initiate signaling cascades, and culminate into a robust inflammatory response to kill and phagocytose the microorganism. PAMPs often cause the initial cascade of proinflammatory signaling in the early stages of illness, and this inflammation can damage nearby cells, thus releasing DAMPs into circulation and initiating a snowball proinflammatory signaling cascade (Hanisch and Kettenmann, 2007). Macrophages recognize gram-negative bacterial endotoxin lipopolysaccharide (LPS) using a receptor complex including toll-like receptor 4 (TLR4), CD14, and complement proteins, and secrete reactive oxygen and reactive nitrogen species to destroy the pathogen and proinflammatory cytokines in order to call nearby microglia to aid in the response (Lund et al., 2006). While this response is beneficial in most cases of infection, high levels of PAMPs and DAMPs also cause immune cells to release dangerously high levels of cytokines. As such, PAMPs, DAMPs, and cytokines have been targeted in recent therapeutics designed to treat sepsis (Gruda et al., 2018).

Microglia, the brain’s resident macrophages, survey the brain and respond to any disturbances in their environment, resulting in morphological and functional changes during peripheral inflammation and infection (Butovsky and Weiner, 2018). As the main immune effector cell within the brain, they play intimate roles in the mechanisms behind sickness behaviors. During peripheral inflammation driven by increased circulating levels of proinflammatory cytokines, microglia express high levels of inducible nitric oxide producing enzymes, which remain elevated for several days. High levels of nitric oxide can result in destruction of bacteria, but also cause apoptosis of nearby neurons (Heneka et al., 1998). Elevated nitric oxide levels have been linked to synaptic loss in a protein kinase-G dependent manner (Sunico et al., 2010). Additionally, complement-mediated synaptic pruning has been implicated in normal brain development, cognitive aging, and neurodegenerative disease (Presumey et al., 2017). Systemic LPS injection has also been shown to have both disruptive and non-disruptive effects on the BBB during and after inflammation (Varatharaj and Galea, 2017).

Various mouse models of endotoxemia have revealed reduction of synapses in the CA1 region of the hippocampus (Moraes et al., 2015; Zhang et al., 2017). Reduced levels of NMDA protein were reported in the CA1 region of two different mouse sickness models (Zhang et al., 2017), while other studies have found reductions in excitatory synapse number in the hippocampus using cecal ligation and puncture (CLP) models (Moraes et al., 2015). Behavioral deficits, including spatial memory tasks, have been found to be microglia-dependent as pre-emptive minocycline treatment reduced neuroinflammation, oxidative stress, and neuronal dysfunction following CLP (Michels et al., 2015, 2017). Additionally, hippocampal-dependent context discrimination memory is impaired in rats following a single peripheral injection of LPS (Czerniawski and Guzowski, 2014). Recent human studies have similarly found long-term cognitive dysfunction in sepsis survivors. Sickness behavior in humans also includes memory impairment (Capuron et al., 1999; Reichenberg et al., 2001) and even very low doses of endotoxins can cause anxiety and depressive symptoms in humans (Reichenberg et al., 2001; Krabbe et al., 2005). Cognitive dysfunction and sickness behavior was exacerbated in aged mice in response to intracerebroventricular LPS administration (Huang et al., 2008). Synaptic loss is considered the best known correlate of cognitive dysfunction, but the direct role of microglia in synaptic loss in sepsis or sickness behavior has not yet been investigated.

To provide insights into this possible involvement, the present study aimed to investigate changes in microglial ultrastructure and interactions with synaptic elements in the *strata radiatum* and *lacunosum-moleculare* of the hippocampal CA1 region, 24 h after peripheral LPS administration in mice. We chose a 24 h timepoint as previous studies have found changes in ultrastructural interactions between microglia and cortical neurons, and disruptions in inhibitory synapses 24 h after peripheral LPS injection (Chen et al., 2014). We focused on the CA1 as it is the main region implicated in spatial memory tasks, where deficits were seen in prior studies in both human cases and mouse models of illness (Michels et al., 2015; Calsavara et al., 2018; Barichello et al., 2019). Our quantitative analysis determined that microglial cell body and process ultrastructure, as well as interactions with the neuropil, including synaptic clefts, were significantly modified following peripheral LPS administration.

## Materials and Methods

### Animal Model

All experimental procedures were performed in agreement with the guidelines of the Institutional Animal Ethics committees, in conformity with the Canadian Council on Animal Care and the Animal Care Committee of *Université Laval*. Animals were group housed three to five animals per cage under a 12-h light-dark cycle at 22–25 °C with free access to food and water. Four month old CX3CR1-GFP heterozygous mice on C57BL/6J background (The Jackson Laboratory) were injected intraperitoneally (i.p.) with saline or 1 mg/kg of LPS derived from *Escherichia coli* serotype O55:B5 (Sigma Aldrich). CX3CR1-GFP mice were used considering that a subset of mice from the same protocol were imaged using two-photon *in vivo* microscopy (Abiega et al., 2016; Paris et al., 2018). The dose and timing of LPS was defined by the minimal dose required to induce sickness behavior while preventing mortality in our and other studies, and coinciding with changes in microglia-neuron interactions in previous studies in mouse cortex (Chen et al., 2014; Hoogland et al., 2015). A small cohort (four saline and five LPS injected animals) were treated to verify this dose and afterward utilized for two-photon *in vivo* microscopy (Abiega et al., 2016; Paris et al., 2018). Following LPS injection, murine sickness score (MSS) was assessed every 2 h as previously described (Shrum et al., 2014) by an observer blinded to the experimental conditions. Coat appearance, level of consciousness, activity, response to stimulus, eye appearance, and respiration rate and quality were assessed every 2 h until 10 h post-injection. Male and female mice were split evenly between groups. Seven saline and seven LPS injected animals were used for open-field behavior studies, keeping only those with an optimal ultrastructural preservation (five saline and six LPS injected animals) for electron microscopy studies.

### Open-Field Test

Twenty four hours after injection, mice were subjected to open-field testing (Hui et al., 2018). Carefully, one mouse was placed at the center of the apparatus (i.e., 50 × 50 cm white laminated cardboard box) and allowed to move freely for ten minutes. The movement was recorded with the ANY-maze system (version 4.8, Stoelting, Wood Dale, IL, United States). The total distance traveled, lines crossed, distance traveled at the center, entrances into the center, body rotations, freezes and immobile episodes were determined. The apparatus was cleaned between each mouse with 70% ethanol.

### Animal Sacrifice and Tissue Processing

Immediately after open-field testing, mice were anesthetized with a cocktail of 80 mg/kg ketamine and 10 mg/kg xylazine. The animals were then transcardially perfused with ice-cold phosphate-buffered saline (PBS; 50 mM at pH 7.4) followed by 3.5 % acrolein and 4% paraformaldehyde (PFA) both diluted in phosphate buffer (PB; 100 mM at pH 7.4). Brains were harvested and post-fixed 2 h in ice-cold 4% PFA. Following post-fixation, brains were washed with PBS to remove excess PFA. Fifty-micrometer thick coronal brain sections were generated in PBS using a vibratome (Leica VT1000s). Brain sections were stored in a solution of cryoprotectant and stored at −20°C (Bisht et al., 2016a).

### Tissue Preparation Staining for Electron Microscopy

Immunohistochemistry was performed against ionized calcium-binding adaptor protein 1 (IBA1) which provides an excellent visualization of microglial fine processes by immunocytochemical electron microscopy, as described previously (Savage et al., 2018). Briefly, brain sections between −2.0 mm and −2.3 mm Bregma levels were selected and washed in PBS to remove cryoprotectant, then incubated in 0.3% hydrogen peroxide followed by 0.1 % sodium borohydride. Sections were incubated 1 h in blocking buffer (10% fetal bovine serum, 3% bovine serum albumin, 0.03% Triton X-100) then overnight at 4°C in primary rabbit anti-IBA1 antibody in blocking buffer (Wako). Sections were next incubated for 90 min in goat-anti-rabbit IgGs conjugated to biotin (1/300, diluted in TBS, Jackson ImmunoResearch) followed by ABC reagent (Vector Laboratories) and developed with a solution containing 0.05% 3,3′-diaminobenzidine and 0.015% hydrogen peroxide. A 30-min incubation of sections with 1% osmium tetroxide to fix lipids was performed followed by an ethanol dehydration of increasing concentration, washing in propylene oxide and overnight infiltration in Durcupan resin. The next day, the sections were embedded with Durcupan resin between ACLAR embedding films (Electron Microscopy Sciences) for 72 h at 55°C.

The CA1 of the dorsal hippocampus was excised, affixed to a resin block and cut into 70–75 nanometer-thick sections using an ultramicrotome (Leica Ultracut UC7). The ultrathin sections were collected on copper mesh grids. In each animal, 10 microglial cell bodies and 150–250 microglial processes were randomly selected and imaged at a magnification of 6800× using a transmission electron microscope (FEI Tecnai Spirit G2) equipped with an ORCA-HR digital camera (Hamamatsu; 10 MP). Microglial cell bodies were identified based on their positive staining for IBA1 and their unique ultrastructure. Microglia generally have smaller cell bodies and nuclei than neighboring astrocytes or neurons, characteristic heterochromatin patterns in their nuclei, as well as long and narrow stretches of endoplasmic reticulum (ER) (Savage et al., 2018). Microglial processes were identified based on their positive staining for IBA1 and their lack of nucleus, ER or Golgi apparatus. Intracellular organelles were identified as previously described (El Hajj et al., 2019).

### Ultrastructural Analysis of Microglia

Images of the microglial cell bodies and processes were blinded to the experimental conditions prior to analysis to prevent bias. The area, perimeter, circularity, solidity, number of phagosomes, percentage of cells with phagosomes and presence of ER dilation was determined for each cell body using FIJI. Additionally, the maximum distance from nuclear membrane to cellular membrane was measured, as well as the number of cells displaying proximal processes. A proximal process was defined as a region which narrows to below 0.3 microns and does not contain ER or Golgi apparatus. Excitatory synapses (asymmetric synapses) were defined by the presence of a presynaptic axon terminal containing 40-nanometer vesicles in close apposition to a postsynaptic dendritic spine displaying an asymmetric postsynaptic density thickening (Colonnier, 1968). The perimeter, area, percentage touching synapses, percentage with phagosomes and percentage associated with extended extracellular space pockets was determined for each process (El Hajj et al., 2019). Phagosomes were identified by their ovoid shape with a clear cytoplasm. ER was characterized as dilated if the distance between the two membranes enclosing the lumen was greater than 60 nanometers. Extracellular space pockets were identified by clear space surrounding the microglia, without delineating membranes and lacking acute angles seen in astrocytic processes (Tremblay et al., 2010).

### Statistics

Data was analyzed using GraphPad Prism 7. LPS versus saline injected mice were compared using a non-parametric Mann–Whitney test. The sample size (n) refers to individual microglial cell bodies or processes, as previously reported in our ultrastructural analyses (El Hajj et al., 2019). All data is reported as mean ± standard error of the mean (SEM).

## Results

**FIGURE 1:**
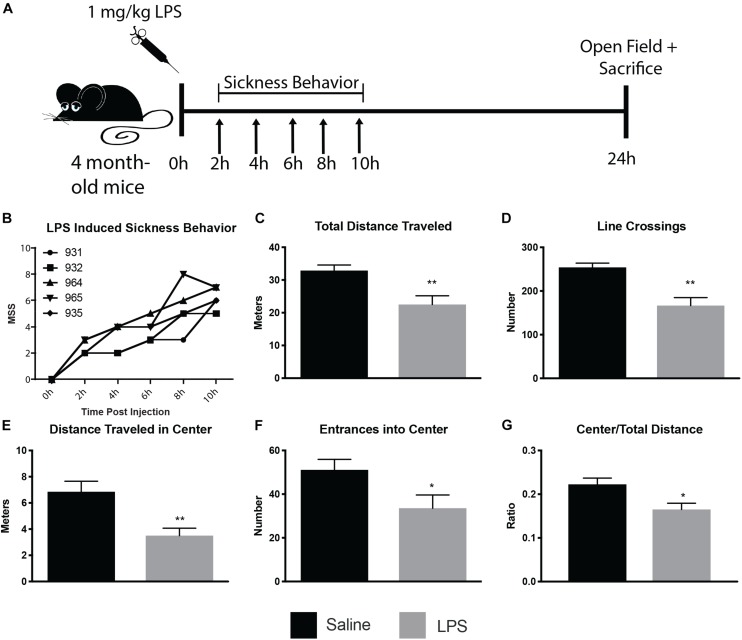
LPS induced sickness behavior in 4 month old mice. Mice were injected with 1 mg/kg LPS or saline and assayed for sickness behavior, using the murine sickness score (MSS) tallied every 2 h for 10 h post-injection and open field behavior assay 24 h post-injection **(A)**. LPS-injected mice displayed increases in MSS **(B)** and decreases in total distance traveled **(C)**, line crossings **(D)**, distance traveled in the center **(E)**, entrances into center **(F)**, and center/total distance traveled **(G)**. ^∗^*p* < 0.05, ^∗∗^*p* < 0.01.

To determine the ultrastructural changes of microglia and their interactions with synaptic structures during sickness behavior, we injected 4 month old CX3CR1-GFP heterozygous mice i.p. with 1 mg/kg LPS or saline (see Figure 1A for experimental paradigm). An observer blinded to animal treatment monitored the injected mice for sickness behavior during 10 h following injection (Figure 1B), and was able to correctly identify LPS from control mice, as control mice displayed a score of 0 on the MSS at every timepoint investigated. Mice injected with LPS displayed time-dependent increases in MSS (Figure 1B). Twenty-four hours after injection, mice were subjected to open-field behavior testing to verify sickness behavior. Mice displayed sickness behavior including decreased distance traveled both in total and in the center of the open-field, and decreases in the number of line crossings (Figures 1C–G), consistent with other rodent sickness behavior models (Shrum et al., 2014; Furube et al., 2018; Mansour et al., 2018).

**FIGURE 2:**
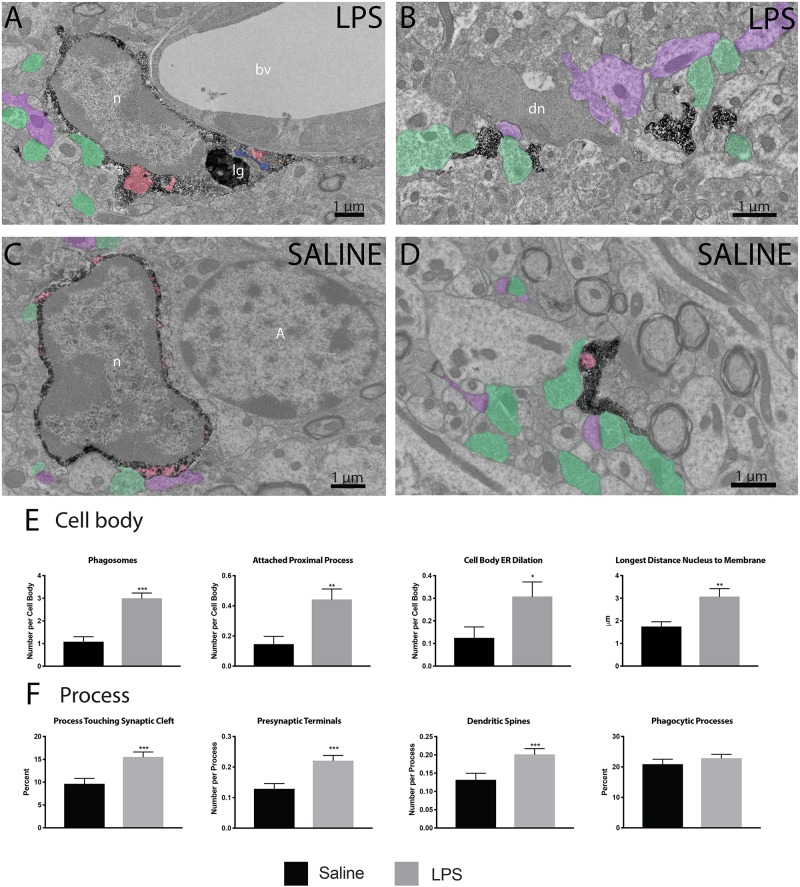
LPS induced variations in microglial ultrastructure. Microglial cell bodies **(A,B)** and processes **(C,D)** within the *strata radiatum* and *lacunosum-moleculare* region of CA1 hippocampus were stained with anti-IBA1 antibody and investigated using transmission electron microscopy. Microglial cell bodies in LPS treated animals often contained long processes connected to their cell bodies, numerous lipidic (lg) and phagocytic inclusions (red) as well as dilated endoplasmic reticulum (ER; blue). Microglial processes often contacted asymmetric synapses including presynaptic terminals (green) and postsynaptic dendritic spines and shafts (purple). In panel **(B)**, a dark dendrite is also seen extending a dark spine that receives a synaptic contact from a healthy-looking axon terminal. The interaction of microglial cell bodies **(E)** was quantified, including number of phagosomes, attached proximal processes, and number of dilated ER stretches per cell body, as well as the longest distance between the cell body and nucleus. The interaction of microglial processes **(F)** with the neuropil was also quantified, including interactions with synaptic structures comprising synaptic clefts, presynaptic terminals and postsynaptic dendritic spines. ^∗^*p* < 0.05, ^∗∗^*p* < 0.01, ^∗∗∗^*p* < 0.001. bv – blood vessel, n – nucleus, lg – lipid granule, dn – dark neuron, A - astrocyte.

Following verification of LPS injection causing sickness behavior, we analyzed microglia at nanoscale resolution to determine changes in ultrastructure and interactions with synapses. We focused on the *strata radiatum* and *lacunosum-moleculare* of the dorsal CA1, which has been implicated in microglial-mediated behavioral deficits in mouse models of sickness behavior (Michels et al., 2015; Zhang et al., 2017). Microglial cell bodies and processes were identified by their immunoreactivity against IBA1, with cell bodies displaying characteristic bean-shaped nuclei, long stretches of ER and numerous mitochondria (Figures 2A–D).

Microglial cell bodies were more likely to contain phagosomes in LPS injected *versus* saline injected animals (84.62 percent versus 43.75 percent, Table 1). LPS injected animals also displayed increased numbers of phagosomes per microglial cell body (Figure 2E). Most of these phagosomes were lucent and contained fully digested contents. However, many phagosomes contained partially degraded membranes and in one case contained what may appear to be two partially digested postsynaptic densities. Cell bodies were also more likely to display attached proximal processes, defined by a narrowing to less than 300 nanometers in width at some point also devoid of ER and Golgi (Figure 2E). Attached proximal processes are very rarely seen in ultrathin sections when investigating healthy brain tissue, but were significantly increased after LPS injection (14.58 percent in saline injected controls *versus* 44.23 percent in LPS injected animals, Table 1). The cytosol of microglial cell bodies from LPS injected animals was also thicker and more expansive compared with saline injected controls, as determined by the longest distance measured between cellular and nuclear membranes (Figure 2E).

**TABLE 1:** Quantification of ultrastructural changes induced by LPS in 4 month old mice.

	**Saline**	**LPS**	***p*-value**
**Cell bodies**			
Area (μm^2^)	20.41 ± 1.11	23.65 ± 1.727	0.116
Perimeter (μm)	22.41 ± 0.88	25.58 ± 1.32	0.0477
Circularity	0.525 ± 0.02	0.482 ± 0.02	0.136
Roundness	0.5591 ± 0.023	0.5733 ± 0.023	0.658
Solidity	0.844 ± 0.014	0.814 ± 0.014	0.126
Phagocytic cells (%)	43.75 ± 7.24	84.62 ± 5.05	< 0.0001
Phagosomes per cell (n)	1.083 ± 0.22	3 ± 0.231	< 0.0001
Lipid bodies (%)	20.83 ± 5.9	21.15 ± 5.7	0.969
Lipid bodies per cell (n)	0.4583 ± 0.1657	0.4615 ± 0.1516	0.988
Extracellular space pockets (%)	56.25 ± 7.2	59.62 ± 6.87	0.7365
ER dilation (%)	12.5 ± 4.8	30.77 ± 6.46	0.0276
Attached proximal processes (%)	14.58 ± 5.15	44.23 ± 6.96	0.001
Cell body cytoplasmic size (μm^2^)	6.724 ± 0.516	10.75 ± 1.11	0.0018
Distance nucleus to membrane (μm)	1.679 ± 0.180	2.956 ± 0.314	0.0002
**Processes**			
Area (μm^2^)	0.278 ± 0.016	0.334 ± 0.017	0.86
Perimeter (μm)	2.49 ± 0.092	2.51 ± 0.073	0.43
Circularity	0.559 ± 0.008	0.589 ± 0.006	0.007
Roundness	0.523 ± 0.008	0.527 ± 0.006	0.725
Solidity	0.826 ± 0.005	0.842 ± 0.004	0.016
Touching synapse (%)	9.646 ± 1.19	15.53 ± 1.08	0.0005
Number of synaptic clefts per process (n)	0.111 ± 0.015	0.173 ± 0.013	0.002
Axon terminals per process (n)	0.129 ± 0.018	0.221 ± 0.017	0.0005
Spines per process (n)	0.133 ± 0.018	0.201 ± 0.016	0.005
Phagocytic (%)	20.9 ± 1.6	22.7 ± 1.25	0.337
Number of phagosomes per process (n)	0.338 ± 0.035	0.430 ± 0.028	0.365
Extracellular space pockets (%)	40.68 ± 1.97	37.89 ± 1.45	0.253

While microglial cell bodies in LPS injected animals contained a larger area of cytosolic space (10.75 μm^2^ versus 6.72 μm^2^, Table 1), they were not significantly different in total area or perimeter. Roughly 60 percent of cell bodies contacted pockets of extracellular space in both conditions (56.25 percent in saline injected *versus* 59.62 percent in LPS injected mice). Previous studies from our group have defined dark microglia, recognized by their electron dense cytoplasm and nucleoplasm, as cells displaying signs of metabolic stress including dilated ER (Bisht et al., 2016b). These cells are rare in healthy young adult mice, but they increase in number among the hippocampus CA1 *strata radiatum* and *lacunosum-moleculare* of maternal immune activation, chronic stress, aging or Alzheimer model mice (Bisht et al., 2016b; Hui et al., 2018). We found no dark microglia in either experimental group, indicating that acute sickness induced by a single LPS injection is an insufficient stressor to induce microglial cytoplasmic/nucleoplasmic condensation, the hallmark identifying ultrastructural feature of dark microglia, at least in this brain region and at the time point examined. While no dark microglia were identified, occasional stressed or degenerating dendritic spines were observed based on their darkened cytoplasm and altered organelles, often in contact with microglial processes (Figure 2B).

In addition to investigating microglial cell bodies, we utilized anti-IBA1 staining to identify at the ultrastructural level microglial processes discontinuous to their cell body in ultrathin section. Because IBA1 is distributed throughout the cytosol, we were able to investigate processes and their interactions with the surrounding neuropil. Although microglial processes did not change size following LPS injection, they were rounder and increased in solidity compared with the saline injected controls (Figures 2B,D). This is in line with light-level analyses of microglia conducted in other sickness behavior models (Hoogland et al., 2015). Microglial processes in LPS injected animals were also significantly more likely to interact with excitatory synapses. In particular, processes from LPS injected animals were much more likely to directly touch synaptic clefts (15.53 percent versus 9.6 percent, Table 1) and interact with both presynaptic axon terminals and postsynaptic dendritic spines (Figure 2F) than those of saline injected controls.

## Discussion

Microglia have been implicated in the initiation of neuronal death and damage across a myriad of neurodegenerative conditions including models of sickness behavior and sepsis survivors (Block et al., 2007; Sankowski et al., 2015; Zhao et al., 2019). We are reporting the first quantitative ultrastructural characterization of microglia within the hippocampus CA1 (*strata radiatum* and *lacunosum-moleculare*) of an LPS induced mouse sickness model. Our results uncovered increases in phagosomes 24 h after peripheral exposure. As we performed all of our TEM analysis on individual ultrathin sections and not serial sections, we cannot rule out the possibility that these phagosomes could be incidences of extracellular digestion or “exophagy” (Haka et al., 2016) viewed in 70–75 nanometer-thick profile. However, the incidence of microglia-associated extracellular space pockets was not significantly different between LPS and saline injected controls. In addition to increases in putative phagosomes, we also detected increased microglial interactions with synaptic structures when observed 24 h after peripheral LPS injection.

Dark microglia have been described in the hippocampus CA1 region of various mouse models in conjunction with disruptions in their inflammatory signaling, including animals subjected to chronic unpredictable stress, repeated social defeat, maternal immune activation, aging, Alzheimer pathology and CX3CR1 deficiency (Bisht et al., 2016b; Hui et al., 2018; El Hajj et al., 2019). While a single dose of LPS was sufficient to induce proinflammatory signaling and significant changes in microglial cell body and process ultrastructure, we found no evidence of dark microglia in the CA1 (*strata radiatum* and *lacunosum-moleculare*) of LPS injected animals. It appears that the acute reaction to a single dose of LPS is insufficient to induce the dark microglia phenotype in 4 month old animals. This could imply that such a shift in microglial phenotype requires chronic increases in inflammatory signaling. Further studies are required to investigate the long-term consequences of LPS induced sickness and recovery on microglial ultrastructure.

While we did not observe dark microglia in the CA1 of LPS injected animals, we identified dark neuronal spines and cell bodies, for the first time in a sickness behavior model. These dark neurons, which could represent a subset of susceptible neurons, were previously described in aged mice and in mice experiencing stressful challenges, including sensory loss (Peters et al., 1991; Tremblay et al., 2012), as well as neurodegenerative disease pathology (Turmaine et al., 2000). Our data is in line with previous studies showing that mice subjected to i.p. LPS display reductions in neuronal projections (microtubule associated protein 2 staining) accompanied by neuronal cell body loss in the hippocampus (Zhao et al., 2019). Additionally, mouse models of sepsis have lower levels of glutamatergic NMDA receptors, as well as reduced numbers of doublecortin-positive newborn neurons and parvalbumin interneurons in the hippocampus (Valero et al., 2014; Ji et al., 2015; Zhang et al., 2017). These alterations were shown to be triggered by inflammatory pathways, most likely through increased microglial secretion of proinflammatory cytokines and reactive oxygen species.

Microglia in LPS injected animals contained increased amounts of phagocytic material. It is well established in the literature that peripheral administration of LPS can cause a shift in microglial morphology toward a more amoeboid shape (Buttini et al., 1996; Hoogland et al., 2015). It is possible that exposing microglia to proinflammatory cytokines shifts their activity to a more pro-phagocytic state. In our study, microglial cell bodies in animals injected with LPS contained nearly three times the phagocytic cargo observed in those injected with saline. These data imply that proinflammatory cytokines, at least in the short term of 24 h, significantly increase microglial phagocytic activity. However, further studies focused on the expression of proinflammatory cytokines, chemokines, and phagocytic cell surface receptors by these cells is required to directly link LPS administration with increases in microglial phagocytosis.

While several models of microglial morphology have implied that more amoeboid cells are associated with cell body migration and phagocytosis at the expense of surveillance (Walker et al., 2014), microglia in LPS injected animals were significantly more likely to interact with excitatory synapses (both elements and clefts). This is not immediately intuitive as microglia were previously defined with phenotypes somewhere on a spectrum between surveillant (interacting with synapses) and reactive (increasing cytokine/chemokine response to invading pathogens followed by phagocytic clearance) (Kreutzberg, 1996; Butovsky and Weiner, 2018). However, microglia involved in the phagocytosis of newborn neurons generated through adult neurogenesis or synapses during normal brain development were previously shown to display a ramified morphology with ‘ball and chain’ structures of phagocytic pouches on ramified processes (Sierra et al., 2010; Tremblay et al., 2010; Schafer et al., 2012). In the context of chronic stress, microglia which are involved in neuronal circuit rewiring (Milior et al., 2016; Wohleb et al., 2018) were either shown to display reduced or hyper-ramified processes depending on the model and time course (Hinwood et al., 2013; Hellwig et al., 2016; Milior et al., 2016). The synaptic contacts we observed did not appear to be sites of active proteolytic degradation, as there was no change in the amount of microglia-associated extracellular debris, marked by partially degraded membranes located in the large pockets of extracellular space between microglial membranes and surrounding neuropil, between either group of microglia. However, several microglial cell bodies displayed putative phagosomes containing seeming intact neuropil, while partially digested membranes and possible postsynaptic densities were also found in LPS injected mice.

Microglia have previously been implicated in synaptic pruning in a cellular mechanism known as “trogocytosis” (Weinhard et al., 2018). Weinhard and colleagues demonstrated that microglial processes in postnatal day 15 animals removed small parts of axon terminals averaging between 0.1 and 0.5 μm^3^, much smaller than traditionally phagocytosed elements, without extracellular space between the neuronal and microglial membrane. It is possible that these excess synaptic contacts in LPS injected animals were undergoing trogocytosis, although further studies using 3-dimensional electron microscopy would be necessary to verify complete engulfment. It is also possible that these processes are engaged in synaptic stripping, by which a microglia remove synapses by interjecting a process into the synaptic cleft, physically separating the presynaptic terminal from the dendritic spine or shaft (Blinzinger and Kreutzberg, 1968). Synaptic stripping has been described in cortical samples from mice sacrificed 24 h after peripheral LPS injection (Chen et al., 2014).

Our study focused on the microglial response 24 h after a single injection of LPS, which has allowed us an ultrastructural snapshot of what occurs inside the brain during recovery from acute sickness. Numerous studies have studied acute (2–6 h post LPS injection) sickness behavior in rodents but have found increases in depressive-like behavior 24 h after injection (Dantzer et al., 2008). Human studies have also uncovered both acute and long-term implications of peripheral inflammation on the emergence of depression and other psychiatric disorders (Savitz and Harrison, 2018). Other studies in rodents have noted various long-term changes to microglial morphology after single or multiple exposures to LPS, being present in some cases as much as a year after injection (Hoogland et al., 2015). As this is the first ultrastructural characterization of microglia in the hippocampus in response to systemic LPS administration we focused on a single timepoint during initial sickness behavior. While we investigated both male and female mice there were no overt differences between the microglial ultrastructure between the two sexes in response to LPS, although the response to LPS administration was rather robust and our sample size was insufficient to detect possibly small changes due to sex differences. In addition to sex differences, there are numerous questions to be addressed in follow-up studies, including exploration of acute *versus* chronic ultrastructural changes of microglia in response to a single dose or multiple doses of LPS, as well as investigation into other brain regions and along the aging trajectory. In complement, it would also be important to use additional techniques than electron microscopy, beyond the scope of this short communication study, to provide cellular and molecular insights into the microglial activities, such as neuronal circuit rewiring, mediated during sickness behavior.

## Data Availability Statement

The datasets generated for this study are available on request to the corresponding author.

## Ethics Statement

The animal study was reviewed and approved by the Université Laval.

## Author Contributions

M-ET obtained funding for the study. JS designed and performed the experiments with CH. JS and M-KS-P prepared and analyzed the electron microscopy pictures and behavioral data. JS wrote the first draft of the manuscript. M-ET edited the subsequent versions to which all authors contributed. All authors read and approved the final version of the manuscript.

## Conflict of Interest

The authors declare that the research was conducted in the absence of any commercial or financial relationships that could be construed as a potential conflict of interest.
